# Host-directed therapeutic targets in macrophages and their ligands against mycobacteria tuberculosis

**DOI:** 10.1128/iai.00063-25

**Published:** 2025-08-25

**Authors:** Jin Li, Yanmei Wang, Lei He, Luchuan Yang, Tao Tao, Lang Bai, Youfu Luo

**Affiliations:** 1 Center of Infectious Diseases and State Key Laboratory of Biotherapy, West China Hospital, West China Medical School, Sichuan University 34753, Chengdu, China; 2 Institute of TCM Clinical Foundation and Literature Information, Sichuan Academy of Chinese Medicine Sciences 598782 , Chengdu, China; 3 Institute of Traditional Chinese Medicine of Sichuan Academy of Chinese Medicine Sciences (Sichuan Second Hospital of T.C.M) 599924 , Chengdu, China; University of California Merced, Merced, California, USA

**Keywords:** host-directed therapies, drug resistance, macrophage polarization, metabolism reprogramming, *Mycobacterium tuberculosis*

## Abstract

Although current combination regimens of antibiotics have significantly improved tuberculosis (TB) cure rates, substantial challenges persist in the global effort to end TB. These include poor patient compliance, the emergence of drug-resistant strains due to prolonged treatments, and the persistence of latent TB infections. Host-directed therapies (HDTs) have emerged as a promising complementary strategy, leveraging the modulation of host immune responses to combat *Mycobacterium tuberculosis* (Mtb). Unlike conventional antibiotics, HDTs can enhance therapeutic outcomes by boosting host defense mechanisms, reducing treatment duration and dosage, and minimizing the risk of resistance development. Notably, several HDTs have shown significant efficacy against multidrug-resistant (MDR) Mtb strains, while also mitigating excessive inflammation and lowering relapse rates—achievements that remain elusive with antibiotic regimens alone. This review provides a comprehensive overview of recent advancements in HDTs, focusing on druggable targets and the mechanisms by which these therapies restore or enhance immune functions disrupted by Mtb. By integrating insights into macrophage polarization, metabolic modulation, autophagy promotion, and cell death regulation, HDTs offer innovative and multifaceted approaches to TB treatment. Furthermore, the potential for HDTs to synergize with existing antibiotics underscores their relevance in overcoming current therapeutic limitations. This synthesis aims to inspire further research and development, with the ultimate goal of advancing HDTs as a transformative solution for TB management.

## INTRODUCTION

Tuberculosis (TB), caused by *Mycobacterium tuberculosis* (Mtb), remains one of the leading infectious diseases with high morbidity and mortality rates (1). According to World Health Organization reports, approximately 6.4 million new TB cases (including about 0.45 million drug-resistant cases) were diagnosed in 2021, with 1.6 million deaths (including HIV-positive individuals) recorded. These figures represent a partial rebound to levels comparable to 2017 (2). This challenging situation is primarily attributed to Mtb’s unique cell surface lipids and secreted protein effectors, which modulate various signaling pathways—either agonistically or antagonistically—to evade immune cells and adapt to the microenvironment (3, 4). Additionally, the emergence of resistant strains and treatment failures, compounded by poor patient compliance with lengthy treatment regimens, has driven researchers to explore novel therapeutic approaches. Among these, host-directed therapies (HDTs) that modulate host-pathogen interactions have gained increasing attention over the past decade (5, 6).

Mtb primarily establishes infection by targeting lung-resident macrophages, with alveolar macrophages serving as the first responders. Upon encountering Mtb, macrophages engulf the pathogen into phagosomes, which ideally mature into degradative organelles following fusion with lysosomes (7, 8). In addition to phagocytosis, autophagy is activated to mediate pathogen degradation. The degraded pathogen components are subsequently presented on MHC class I and II molecules, triggering adaptive T-cell responses that further eliminate Mtb (6). However, compared to phagocytosis, the precise role of autophagy in combating Mtb infection has long been debated. Two recent studies have reignited interest in autophagy as a promising therapeutic target (9, 10). These studies reexamined the roles of canonical autophagy and related non-canonical processes, demonstrating that autophagy can prevent Mtb infection in human and mouse cells, as well as in murine models (9, 10).

During infection, the fate and function of macrophages are shaped by various microenvironmental signals within host cells, which play dual roles in infection control. Macrophages are generally expected to shift toward pro-inflammatory phenotypes, which are considered effective against Mtb (11, 12). However, excessive inflammation can result in tissue damage. Apoptosis of infected macrophages aids Mtb clearance by encapsulating the pathogen, thereby facilitating additional defense mechanisms. Conversely, Mtb promotes necrosis in infected macrophages, enabling its dissemination (6). HDTs can mitigate the Mtb burden by modulating host immune cells’ antimicrobial activities—either by blocking host pathways altered by Mtb for its survival or by enhancing protective immune responses that eliminate the pathogen (13). These mechanisms not only reduce treatment duration, antibiotic dosage, and associated toxicity but also decrease the likelihood of Mtb developing resistance (6). Furthermore, HDTs that limit excessive inflammatory responses can help prevent tissue damage. Some HDTs have shown significant promise against multidrug-resistant (MDR) strains and non-replicating Mtb (13). Interestingly, HDTs targeting macrophages also contribute to the antibacterial activity of traditional anti-TB drugs such as isoniazid and pyrazinamide, as well as the recently approved drug bedaquiline (14, 15).

**Fig 1:**
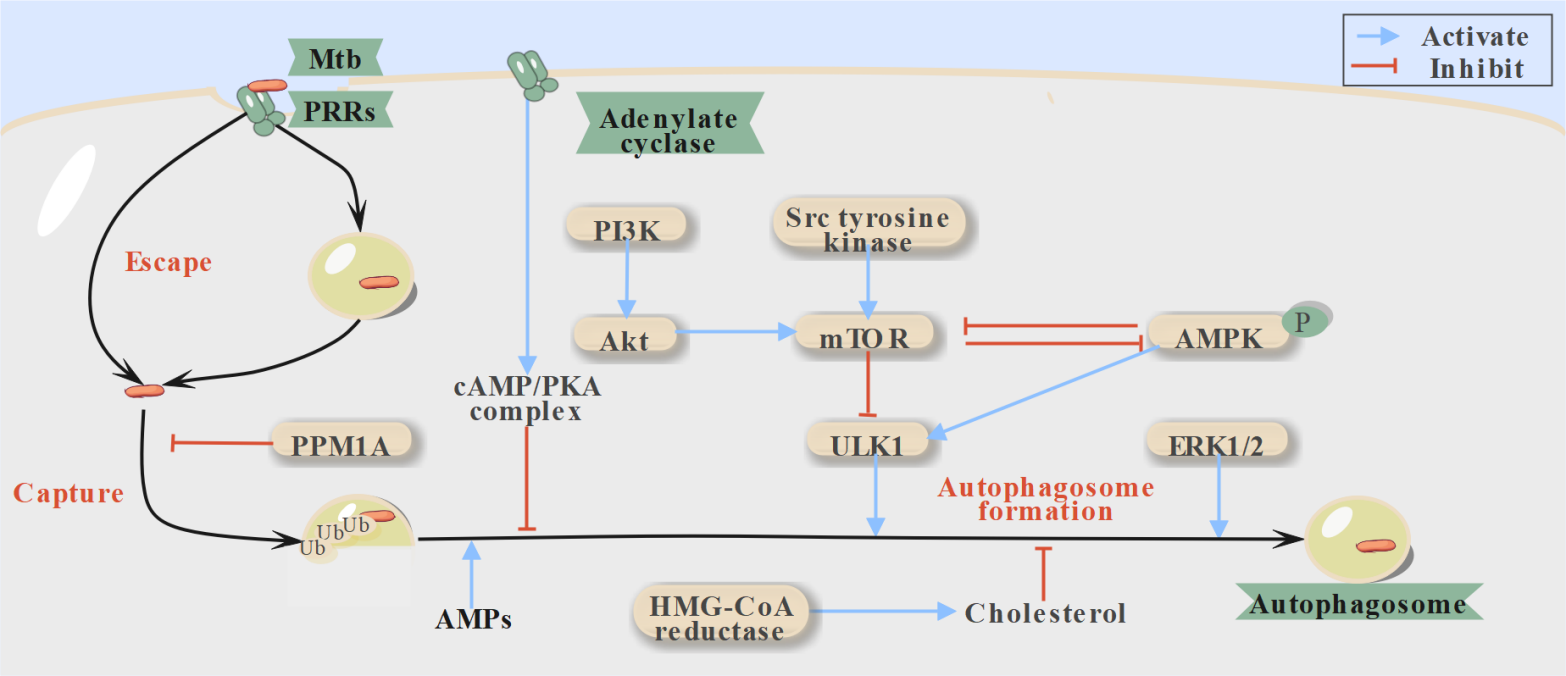
Host-directed therapies (HDTs) targeting autophagosome formation. Escaped Mtb within macrophages can be recaptured through ubiquitination, leading to autophagosome formation and subsequent fusion with lysosomes for degradation. AMPK, AMP-activated protein kinase; AMPs, antimicrobial peptides; mTOR, mammalian target of rapamycin; PKA, protein kinase A; PPM1A, protein phosphatase Mg^2+^/Mn^2+^-dependent 1A; PRRs, pattern recognition receptors; Ub, ubiquitin; ULK1, unc-51-like kinase 1.

**Fig 2:**
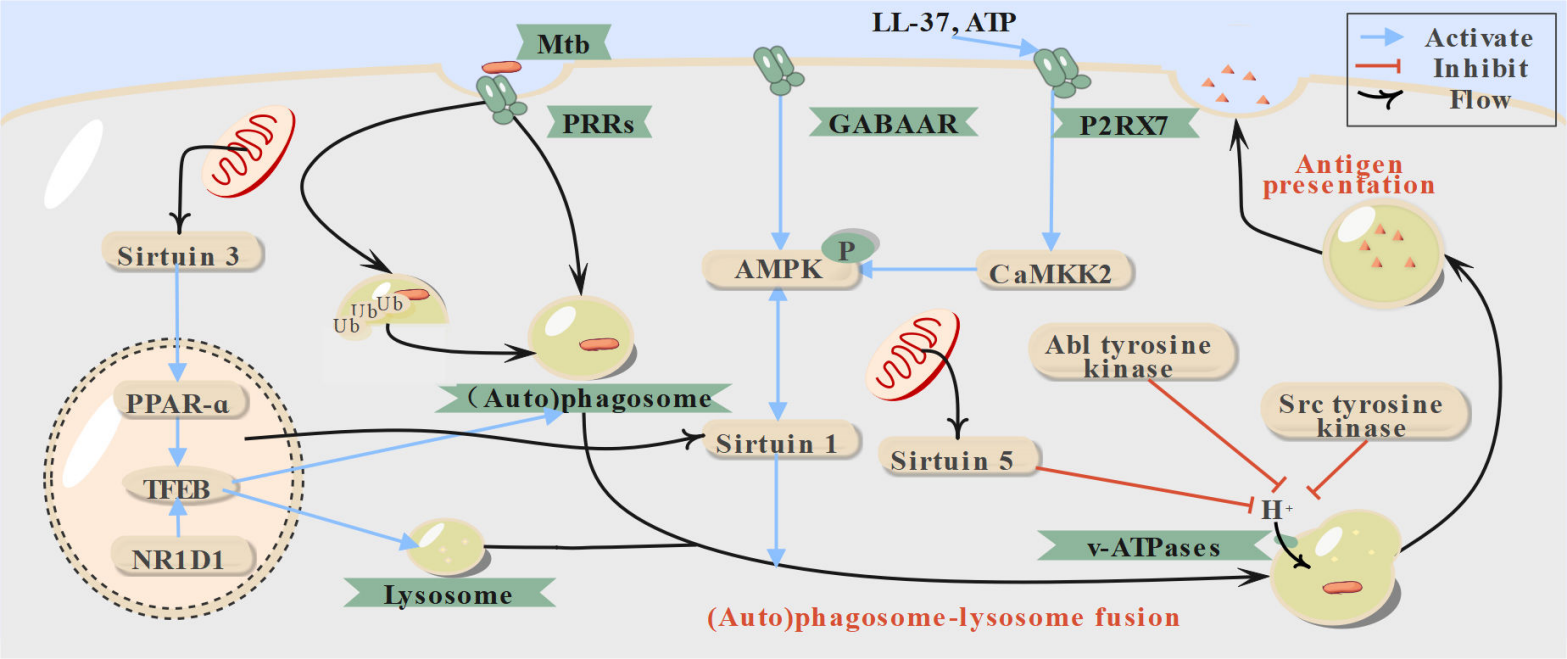
Host-directed therapies (HDTs) targeting other processes during autophagy. Upon infection, Mtb is internalized in the phagosome, the organelle responsible for routine clearance of pathogens. Similar to autophagosomes, phagosomes experience subsequent fusion with lysosomes for degradation. AMPK, AMP-activated protein kinase; CaMKK2, calmodulin-dependent protein kinase kinase 2; GABAAR, type A gamma-aminobutyric acid receptor; LL-37, leucine-leucine-37; mTOR, mammalian target of rapamycin; NR1D1, nuclear receptor subfamily 1, group D, member 1; PPAR, peroxisome proliferator-activated receptor; PRRs, pattern recognition receptors; TFEB, transcription factor EB; Ub, ubiquitin; ULK1, unc-51-like kinase 1; v-ATPases, vacuolar (H+)-ATPases.

**Fig 3:**
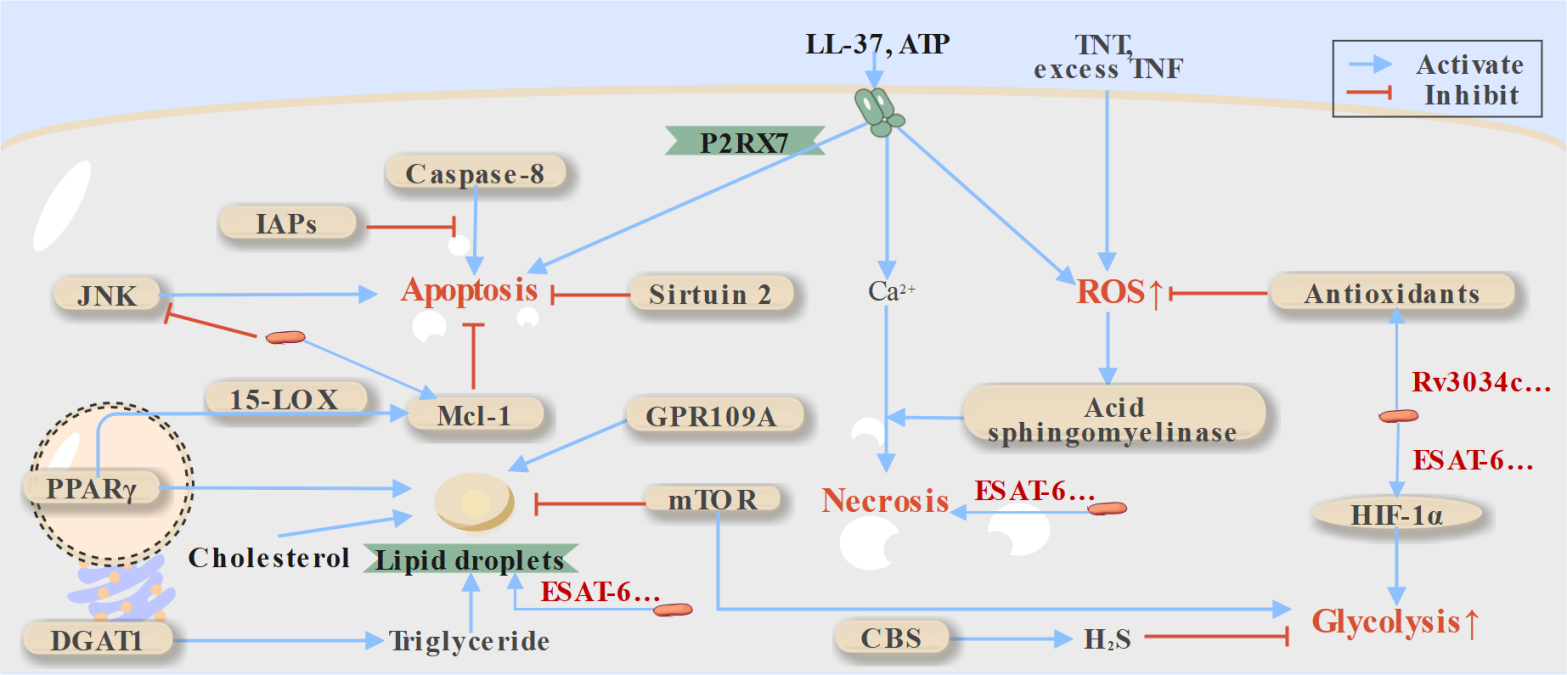
Host-directed therapies (HDTs) targeting metabolic changes during phenotypic variation of macrophages and cell death regulation in macrophages. During TB infection, macrophages exhibit dynamic phenotypic plasticity in response to infection-driven immunometabolic reprogramming and epigenetic modifications. This plasticity manifests as two predominant subtypes: (i) lipid-laden foamy macrophages, characterized by reprogrammed lipid metabolism that promotes excessive lipid droplet accumulation, and (ii) pro-inflammatory M1-polarized macrophages, exhibiting enhanced glycolytic flux and elevated reactive oxygen species (ROS) production to sustain antimicrobial responses. Besides, Mtb infection further triggers divergent cell death pathways, which HDTs may modulate to restore host defense. CBS, cystathionine γ-lyase; DGAT1, diacylglycerol-O-acyltransferase 1; HIF-1ɑ, hypoxia inducible factor-1ɑ; IAPs, inhibitor of apoptosis; JNK, c-Jun N-terminal kinase; 15-LOX, 15-lipoxygenase; mTOR, the mammalian target of rapamycin; PPAR, peroxisome proliferator-activated receptor; TNF, tumor necrosis factor; TNT, tuberculosis necrotizing toxin.

This review focuses on HDTs that modulate key macrophage processes, particularly those involving approved drugs. The availability of safety data and established tissue distribution for these drugs can significantly reduce the time and cost of their application in TB treatment. Additionally, we discuss drugs with efficacy against MDR strains. The relevant targets and their associations are detailed in Tables S1 and S2 ; Fig. 1 to 3.

## HDTs TARGETING PHAGOCYTOSIS AND AUTOPHAGY ENHANCEMENT

### Autophagy: enhancing host defenses against mycobacterial evasion

If the phagocytosis process proceeds as expected, Mtb and their secreted virulent proteins within infected macrophages should be eliminated (Fig. 2) (16). However, virulence factors of Mtb can help them escape from phagosomes into the cytosol by breaking the phagosomal membrane and creating shelters for themselves by suppressing phagosomal maturation (Fig. 1 and 2) (17, 18). Fortunately, some escaped Mtb can be recaptured via xenophagy (host selective autophagy of foreign pathogens) (Fig. 1) (19–21). Intriguingly, Mtb has evolved strategies of modulating host miRNAs or employing protein effectors to subvert this process (22). Beyond directly eliminating bacteria, autophagy also regulates cell death by limiting ESX-1-mediated phagosome disruption and cytosolic access. This regulatory role is evidenced by the dramatic shift in the death pattern of Mtb-infected cells from apoptosis to necrosis following autophagy deletion (9). Notably, restoring autophagy during infection also helps inhibit pyroptosis, a type of cell death that induces inflammation (23).

Unc-51-like kinase 1 (ULK1), a key autophagy initiator involved in autophagosome formation, has also been implicated in promoting cholesterol efflux. This activity suppresses Mtb growth by reducing lipid accumulation, which is crucial for the formation of foamy macrophages—cells thought to contribute to the long-term persistence of Mtb (24, 25). However, ULK1’s function can be inhibited by the mammalian target of rapamycin (mTOR) and activated by adenosine monophosphate-activated protein kinase (AMPK) (Fig. 1), highlighting both as promising HDT targets for TB treatment (26). Additionally, modulating P2RX7 and transcription factor EB (TFEB, a key regulator of numerous autophagy-related genes), both of which play vital roles in autophagy, has been suggested as a feasible strategy to reduce Mtb bacterial load (27, 28).

#### mTOR inhibition

The mTOR is a central regulator of cell growth in host cells, and its robust activation has been widely observed during Mtb infection, aiding the pathogen’s survival (29). Rapamycin, a well-known mTOR inhibitor, has demonstrated its ability to promote phagosomal maturation and autophagy, reduce inflammation and granulomas (30), and even restore lung tissue regeneration (31). However, the use of rapamycin is limited by its incomplete inhibition of mTORC1 and its lack of effect on mTORC2, which leads to feedback loop-mediated activation (30). Furthermore, sole inhibition of mTORC1 has been shown to increase Mtb load in host cells co-infected with HIV, potentially due to the suppression of phagosomal maturation caused by mTOR inhibitors (32). As a result, future studies on HDTs targeting mTOR should focus on dual mTORC1/2 inhibitors, and it may be necessary to combine these with drugs that promote phagosomal maturation when using autophagy-enhancing therapies.

Among dual mTORC1/2 inhibitors, everolimus has shown direct toxicity to Mtb and the ability to kill intracellular Mtb, even within granulomas. It exerts its effects indirectly by inducing autophagy via mTOR inhibition, stimulating reactive oxygen species (ROS) production, and potentially downregulating lipid droplet formation in THP-1 macrophages (33–36). A phase 2 clinical trial indicated that everolimus treatment over 180 days could improve impaired forced expiratory volume in patients with good safety and tolerability profiles (37). Notably, the emergence of dual-binding site inhibitors like RapaLink-1 offers a solution to resistance issues and enhances antimicrobial efficacy by simultaneously targeting mTOR and other HDT-related pathways against TB (38). These findings underscore the potential of mTOR inhibitors as promising HDT drugs.

Additionally, the Akt/mTOR signaling pathway has been identified as a critical negative regulator of autophagy (Fig. 1). This has been demonstrated by the increased autophagy flux induced by ibrutinib and bazedoxifene through suppression of this pathway (39, 40). Combining these kinase antagonists with mTOR inhibitors is considered a viable strategy to mitigate the potentially undesirable outcomes caused by feedback mechanisms associated with single-inhibitor therapies (41).

#### AMPK activation

Additionally, AMPK, a heterotrimeric serine/threonine protein kinase that activates catabolic pathways, is upregulated to enhance host defense responses during infection. Beyond inhibiting mTOR through the phosphorylation of tuberous sclerosis complex 2 and the regulatory-associated protein of mTOR, AMPK directly activates ULK1, initiating autophagy (Fig. 1) (42). Interestingly, activated AMPK has also been shown to indirectly activate sirtuin 1 by increasing the NAD+/NADH ratio, which subsequently enhances autophagosome-lysosome fusion and further boosts autophagic flux (43). While AMP-mimetic AMPK activators such as 5-aminoimidazole-4-carboxamide-1-β-d-ribofuranoside directly activate AMPK (1), many drugs achieve AMPK activation indirectly by increasing the AMP/ATP ratio, thereby aiding in the elimination of intracellular Mtb. For example, carbamazepine reduces ATP production by limiting mitochondrial Ca^2+^ entry through inositol (1,4,5)-trisphosphate depletion (44). Metformin, on the other hand, utilizes diverse mechanisms to exert antimicrobial effects against both antibiotic-susceptible and MDR Mtb strains. Besides activating AMPK and inhibiting mTOR to promote autophagy, metformin increases superoxide dismutase activity, enhancing isoniazid efficacy while reducing excessive ROS, which could otherwise cause tissue damage. The benefits of metformin alone or in combination with isoniazid or ethionamide in reducing Mtb load and inflammation have been extensively documented. Clinical trials have reported that metformin treatment in patients with diabetes mellitus reduces latent TB reactivation, TB mortality, and improves sputum culture conversion rates (45–47). However, the impact of combining metformin with RHZE therapy on TB treatment is still under investigation (48).

In addition to indirectly activating AMPK via ATP intervention, phosphorylation of AMPK by Ca2+/calmodulin-dependent kinase kinase II (CaMKK2) offers another pathway (Fig. 2). Through this mechanism, activation of macrophage type A gamma-aminobutyric acid (GABA) receptors by GABA or its analogs increases autophagic flux, aiding in the control of Mtb infections (Fig. 2) (49). Furthermore, activation of these receptors has been shown to inhibit pro-inflammatory cytokine production during Mtb infection, further contributing to infection control (49).

#### P2RX7 activation, TFEB upregulation, and autophagy modulators

In addition to the two extensively studied targets for HDTs against TB, activation of the purinergic receptor P2RX7 has been reported to increase levels of ROS and cytosolic free Ca²^+^, as well as enhance the activation of AMPK and the PI3K pathway (Fig. 2) (27, 28). These effects collectively improve autophagic flux. P2RX7 activation also contributes to the antimicrobial activity of leucine-leucine-37, an antimicrobial peptide derived from cathelicidin (Fig. 2). Like other antimicrobial peptides, leucine-leucine-37 directly restricts pathogen survival and can be induced by various drugs, including vitamin D (50), phenylbutyrate (28), and dehydroepiandrosterone (51). Among these, vitamin D has been widely studied. Its use, either alone or in combination with 4-phenylbutyrate, has shown some improvement in TB patients undergoing the RHZE regimen, although its exact benefits remain under debate (52–54). These drugs, including vitamin D, activate P2RX7 indirectly, and an earlier study suggested that micromolar concentrations of certain lipids could enhance the potency of P2RX7 agonists (55), offering an intriguing alternative strategy worth exploring. However, due to the extensive functional repertoire of P2RX7, its effects can vary based on factors such as the intensity and duration of the agonist stimulus, leading to both positive and negative outcomes (56). These considerations should be carefully evaluated before the clinical application of P2RX7 agonists.

Upregulation of TFEB also presents a feasible strategy against TB. Drugs such as ambroxol, tamoxifen, peroxisome proliferator-activated receptor α (PPARα) activators, nuclear receptor subfamily 1 group D member 1 (NR1D1) agonists like GSK4112, PPARα inducers like GW7647, and sirtuin 3 activators like honokiol have shown antimicrobial activity against Mtb. Tamoxifen, in particular, has demonstrated a synergistic effect with rifampin against some MDR strains (57–62). Additionally, extracellular signal-regulated kinase 1/2 (ERK1/2) activators such as pasakbimin A and small molecule inhibitors of human protein phosphatase Mg²^+^/Mn²^+^-dependent 1A (PPM1A), including SMIP-30, promote autophagy by enhancing autophagosome formation and facilitating the targeting of escaped Mtb to autophagosomes, respectively (63–65). Another study highlighted that loperamide significantly reduced Mtb burden in macrophages by promoting autophagy, though its primary target remains under investigation (66).

### Phagocytosis and (auto)phagosomal maturation: overcoming Mtb-induced phagocytosis and acidification blockade

Phagocytosis is facilitated by various pattern recognition receptors present on both immune and non-immune cells (67). After the internalization of Mtb or recapture of escaped Mtb, phagosomes and autophagosomes are both expected to undergo maturation, a process primarily involving phagosome-lysosome fusion and acidification (Fig. 1 and 2) (8). Hence, before enhancing autophagy, the blockade of autophagosome-lysosome fusion caused by Mtb must be addressed (32).

Currently, most HDTs targeting Mtb growth through the promotion of phagosome-lysosome fusion remain in preclinical studies. These include adenylate cyclase inhibitors, protein kinase A (PKA) inhibitors, and sirtuin 1 activators. The former two function by disrupting the c-AMP/PKA complex, which otherwise inhibits fusion by arresting actin assembly (Fig. 1). Sirtuin 1 activators, on the other hand, regulate the expression of related genes and activate AMPK (68, 69). Additionally, gefitinib, an epidermal growth factor receptor inhibitor used for advanced non-small-cell lung cancer, has been reported to restrict Mtb growth by increasing lysosomal biogenesis and modulating cytokine signaling in macrophages, though its primary target remains under investigation (70).

The fusion of phagosomes and lysosomes is closely followed by intravesicular acidification, which enhances the activity of pathogen-degrading enzymes and aids in infection control. However, Mtb counters this process through factors such as mannosylated lipoarabinomannan and virulent proteins like PknG, leading to phagosomal maturation arrest (17, 18). Additionally, during infection, sirtuin 5-mediated expression of cytokine-inducible SH2-containing protein promotes the degradation of catalytic subunit A in vacuolar (H^+^)-ATPases via proteasomes (Fig. 2) (71). Thus, inhibiting sirtuin 5 can facilitate phagosomal acidification, forming the basis of the antimicrobial effects of drugs like pimozide and fluspirilene (67). Enhanced phagosomal acidification also contributes to the antibacterial activity of Abl tyrosine kinase inhibitors against Mtb. For instance, imatinib has shown promising outcomes in reducing granulomas caused by both antibiotic-susceptible and rifampicin-resistant *Mycobacterium marinum*, a species closely related to Mtb and known to induce similar host immune responses (72, 73). Furthermore, intracellular calcium ion levels play a critical role in phagosomal maturation by activating Ca^2+^-calmodulin-dependent protein kinase II (Fig. 2), a process linked to phosphatidylinositol 3-kinase, although the specific mechanism remains unclear (74). This role of calcium is corroborated by the efficacy of calcium modulators in reducing Mtb, with flunarizine exhibiting activity against both H37Ra and H37Rv Mtb strains. However, the detailed molecular mechanisms of drugs like berbamine remain under investigation (75, 76).

In addition to the aforementioned drugs, statins, which inhibit HMG-CoA reductase, have demonstrated antimicrobial activity against Mtb, including the reduction of intracellular Mtb load and TB relapse rates. Macrophages from patients treated with statins exhibit greater resistance to Mtb compared to those from healthy donors without familial hypercholesterolemia (77). Further studies revealed that statins promote phagosomal maturation, enhance autophagy, and modulate inflammation, collectively limiting the growth and spread of Mtb. These effects are associated with reduced total and membrane cholesterol levels in host macrophages (77, 78). Among commonly used statins, simvastatin, fluvastatin, and pravastatin have shown the most significant effects in reducing Mtb loads, while atorvastatin and mevastatin exhibited no effects at nontoxic doses (78). Additionally, reducing vascular endothelial growth factor, a secondary effect of some statins, may further control infection by limiting Mtb dissemination to other organs (79).

Besides the approved drugs mentioned above, Src kinase inhibitors also promote phagosomal acidification. Persistent Src kinase activation leads to mTOR activation via PI3K/Akt phosphorylation, which induces autophagy while simultaneously enhancing phagosomal acidification (Fig. 1 and 2). Notably, AZD0530, a Src kinase inhibitor, has demonstrated efficacy in reducing granulomas caused by various drug-resistant Mtb strains, such as MYC431. However, its relatively high cytotoxicity at elevated doses remains a concern (80–82).

## HDTs TARGETING METABOLIC CHANGES DURING PHENOTYPIC VARIATION OF MACROPHAGES

While phagolysosomal targeting and autophagic flux enhancement constitute primary defense arsenals against Mtb, the pathogen’s remarkable capacity to subvert macrophage polarization necessitates parallel strategies exploiting cellular plasticity (1, 7). Traditionally, macrophages have been categorized into two main types: M1 (classically activated), with pro-inflammatory properties, and M2 (alternatively activated), with anti-inflammatory characteristics. Generally, M2 macrophages facilitate Mtb survival, while pro-inflammatory M1 macrophages inhibit Mtb growth (83).

Notably, the inflammatory stimuli generated by M1 macrophages shift the metabolic profile of unpolarized macrophages from mitochondrial oxidative phosphorylation to aerobic glycolysis, a phenomenon known as the Warburg effect. Additionally, M1 macrophages enhance adaptive immune responses by strengthening antigen presentation (84, 85). However, over time, a transition from the M1 to M2 phenotype has been observed in both humans and mice exposed to Mtb (7, 86).

### Macrophage polarization: rewiring polarization circuits

Although macrophages are expected to adopt an M1 polarization state during infection to combat pathogens, Mtb can induce a biased M2 polarization to evade host immune defenses. This process is mediated in part by ESAT-6, which plays a significant role in promoting M2 metastasis (87). Consequently, influencing macrophage polarization represents a promising strategy for restricting Mtb growth.

During infection, the PI3K/Akt signaling pathway is activated to support Mtb survival in macrophages by inhibiting autophagy and promoting the degradation of the FOXO3 transcription factor. FOXO3 normally suppresses the expression of IL-10, a cytokine that drives M2 polarization. As a result, inhibitors of PI3K and Akt have shown the potential to kill intracellular Mtb by reducing IL-10 expression levels (88). Additionally, antagonism of leukocyte immunoglobulin-like receptor B has been shown to reprogram myeloid-derived suppressor cells toward the M1 macrophage phenotype, underscoring another novel therapeutic target for eliminating Mtb (89).

Another HDT strategy involves promoting M1 polarization through the p38 MAPK signaling pathway. This mechanism has been identified as the key action of biapenem, a widely used carbapenem, and withaferin A, a bioactive steroidal lactone derived from *Withania somnifera*. Both drugs synergized with rifampin and isoniazid, respectively, and reduced TB relapse rates. Notably, withaferin A demonstrated significant reductions in bacterial burdens of MDR Mtb strains in the lungs and spleens of infected mice (90, 91). Additionally, SQ109, a promising antitubercular agent targeting Mmpl3, was found to mediate pro-inflammatory responses via the p38 MAPK pathway (92). Rocaglates also leverage this pathway by sensitizing macrophages to low concentrations of IFN-γ, a cytokine that induces M1 polarization, while simultaneously blocking ERK1/2 phosphorylation and Myc translation, the latter being essential for M2 polarization (93). Recent research has identified activating transcription factor 2 (ATF2), a major dimerization partner of c-Jun, as a potential HDT target. Overexpression of ATF2 sensitized macrophages to M1 polarization stimuli and enhanced adaptive immune responses. Additionally, an auto-regulatory feedback loop between ATF2 and PPM1A was discovered, suggesting another strategy against Mtb through PPM1A inhibition, beyond its role in targeting escaped Mtb to autophagosomes as previously mentioned (94).

In addition to drugs inducing M1 polarization, some compounds improve macrophage antimicrobial abilities regardless of phenotype. These include inhibitors of poly (ADP-ribose) polymerase (PARP) and HDACs. PARP inhibitors, such as rucaparib and niraparib, reduced intracellular bacterial loads of antibiotic-susceptible and MDR Mtb strains in infected macrophages by altering cytokine/chemokine expression and surface markers. However, they paradoxically increased Mtb burdens in M2 macrophages derived from peripheral blood mononuclear cells of healthy donors, highlighting the variability in human macrophage responses that should be considered in future research (95). Regarding HDACs, inhibitors like valproic acid and suberoylanilide hydroxamic acid enhanced the potency of rifampin and isoniazid against intracellular Mtb in combined treatments lasting three days. Valproic acid also showed direct efficacy against extracellular Mtb (96). Other HDAC inhibitors with antimycobacterial effects remain in preclinical trials and have been recently reviewed by both the Heemskerk and Sonawane groups (97, 98). Additionally, phenylbutyrate exerts antimycobacterial effects partly by modulating macrophage polarization, in addition to its ability to activate autophagy in a leucine-leucine-37-dependent manner (28, 52).

### Metabolic checkpoints: fueling antimicrobial immunity

M1 macrophages primarily rely on ATP generated through glycolysis (11, 98), a key metabolic pathway that limits Mtb survival by promoting inflammatory responses. This shift in metabolism, known as the Warburg effect, is mediated by hypoxia-inducible factor-1ɑ (HIF-1ɑ) (11, 12). Compounds that enhance this metabolic shift can strengthen host defenses against Mtb, such as HIF-1ɑ agonists like CoCl_2_ (99) and inhibitors of cystathionine γ-lyase, the enzyme responsible for producing hydrogen sulfide, a gas that suppresses glycolysis (Fig. 3) (100, 101). During this metabolic shift, levels of certain antimicrobial compounds increase, including lactate, the end product of glycolysis, and tricarboxylic acid (TCA) cycle intermediates like succinate. Enzymes involved in glycolysis and the TCA cycle, as well as sirtuins that mediate metabolic regulation via histone deacetylation during infection, are also considered potential HDT targets against Mtb (86).

Additionally, oxidative stress caused by ROS and reactive nitrogen species (RNS) is stimulated to kill intracellular Mtb through mechanisms such as autophagy and cell death (87). However, Mtb counteracts these processes by expressing proteins like Rv3034c and producing antioxidant enzymes to detoxify ROS and RNS (102–106). Targeting these antioxidants with drugs, as summarized in Table S2, presents a promising strategy by inhibiting enzymes involved in redox reactions during infection (107–111). It is worth noting that the Warburg effect may be most prominent during the early phase of infection (87), suggesting that the timing and duration of HDT interventions based on metabolic shifts are critical. During infection, Mtb also reprograms the lipid metabolism by modulating lipid lipolysis in macrophages, leading to a foamy cell phenotype characterized by lipid droplets rich in triglycerides and cholesteryl esters. These lipid droplets are believed to contribute to the long-term persistence of Mtb *in vivo* and the formation of cavities through multiple mechanisms (24). Reducing lipid accumulation by inhibiting enzymes responsible for lipid droplet formation is a feasible anti-TB strategy: Mepenzolate bromide (GPR109A antagonist) and T863 (diacylglycerol-O-acyltransferase 1 inhibitor) have demonstrated certain antimicrobial effects (Fig. 3) (112, 113). Similarly, cholesterol absorption inhibition by ezetimibe effectively reduces lipid accumulation and controls Mtb growth (114). Conversely, fatty acid oxidation exerts a macrophage polarization-dependent regulatory effect on lipid droplet accumulation during Mtb infection. The inhibition of fatty acid oxidation in the M1 phenotype has been believed to control Mtb by eliciting ROS production from the electron transport chain (115–117). But in the M2 phenotype, its inhibition with etomoxir is found to promote the formation of foamy macrophages, which are filled with lipid bodies and impair the control of Mtb intracellular growth (115, 118).

Notably, some studies question the role of lipid droplets in infection control, proposing that they may support the production of protective lipid mediators derived from fatty acids in Mtb-infected macrophages (24, 119). This suggests that further research is needed to fully understand their role and optimize strategies targeting lipid metabolism.

## HDTs TARGETING CELL DEATH REGULATION

Beyond cellular phenotypic adaptation and metabolic remodeling, the ultimate fate of infected macrophages through regulated cell death pathways constitutes a critical third dimension of host-pathogen interactions. Cell death plays a crucial role during Mtb infection and is primarily categorized into apoptosis and necrosis. Apoptosis helps eliminate infected host cells, facilitating T-cell cross-priming while avoiding excessive inflammatory responses. In contrast, necrosis causes tissue destruction, fibrosis, and vascular insufficiency, which contributes to the spread of Mtb, increased drug resistance, and higher rates of treatment failure (19). Accordingly, strategies that promote apoptosis and inhibit necrosis hold significant promise as HDTs. Notably, while pyroptosis and ferroptosis, two recently characterized programmed cell death modalities during TB, have emerged as critical regulators of infectious outcomes, their therapeutic targeting remains largely unexplored in the context of TB.

### Apoptosis

Mtb suppresses apoptosis by inactivating the c-Jun N-terminal kinase (JNK) pathway and inducing pro-survival proteins such as Mcl-1 (120, 121). This makes JNK activation and Mcl-1 inhibition promising strategies for HDTs aimed at enhancing T-cell cross-priming through apoptosis induction. The JNK activator anisomycin has demonstrated the ability to increase apoptosis in infected macrophages and enhance the antimicrobial efficacy of rifampicin (121).

Additionally, Mcl-1 expression during infection is upregulated via 15-lipoxygenase-dependent PPARγ activity (Fig. 3) , highlighting the potential of targeting Mcl-1 and 15-lipoxygenase as HDTs (120). Other apoptosis-based HDT strategies include inhibiting sirtuin 2 and inhibitor of apoptosis proteins (IAPs), both of which promote apoptosis (Fig. 3) (122, 123). Furthermore, P2RX7, a promising target also involved in autophagy promotion, has been shown to induce apoptosis upon activation by ATP (124). However, this mechanism requires further validation with specific activators.

### Necrosis

In addition to inhibiting apoptosis, virulent Mtb factors stimulate macrophage necrosis to promote dissemination to neighboring cells. Cyclophilin-D, a component of the mitochondrial permeability transition pore, has been implicated in necroptosis mediated by acid sphingomyelinase. This lysosomal enzyme is activated by excess ROS generated in macrophage mitochondria following stimulation with excessive tumor necrosis factor (TNF) or TB necrotizing toxin (125, 126). Targeting this pathway by inhibiting cyclophilin-D or acid sphingomyelinase represents a potential strategy to suppress macrophage necroptosis. However, current studies have only demonstrated these effects in *M. marinum* (127).

On another front, phosphodiesterase inhibitors have shown varying effects on TB treatment. Phosphodiesterase-3 inhibitor cilostazol and phosphodiesterase-5 inhibitor sildenafil were found to decrease TNF-α levels by raising cAMP levels, significantly shortening treatment duration when combined with RHZE in murine models of TB (128, 129). Conversely, phosphodiesterase-4 inhibitors exhibited opposing effects in similar models (129, 130). Furthermore, phosphodiesterase-5 inhibitors also reduced Mtb burden by mitigating the immunosuppressive activity of myeloid-derived suppressor cells through cGMP elevation (131). However, it is important to note that anti-TNF therapy has been associated with a high incidence of TB (132), highlighting the need for further exploration of TNF’s dual roles during Mtb infection.

### Ferroptosis

Unlike apoptosis and necrosis, the ferroptosis pathway, a novel iron-dependent form of programmed cell death, has been suggested to facilitate Mtb spread through lipid peroxidation (133). Consistent with this, the lipid peroxidation inhibitor ferrostatin-1 successfully reduced bacterial loads in the lungs and spleens by inhibiting ferroptosis, offering another promising HDT approach for TB treatment (134).

### Pyroptosis

In contrast, pyroptosis, a caspase-1-dependent inflammatory cell death, demonstrates paradoxical roles during Mtb infection. It releases cellular contents directly, which increases immunity but inevitably releases some surviving Mtb, resulting in the expansion of infection scope (135). Current therapeutic interventions targeting pyroptosis remain ambivalent: although few drugs have demonstrated anti-pyroptotic effects during TB, their ability to control Mtb growth remains unclear (136–138). Notably, the combinatorial regimen of *Mycobacterium indicus pranii* and human beta defensin-2 has shown efficacy in curbing Mtb replication by enhancing pyroptosis (139). Consequently, the development of HDTs on pyroptosis must balance optimizing immune-potentiating capacity and circumventing collateral tissue damage, which demands the refinement of the mechanisms that Mtb regulates pyroptosis (135).

## DISCUSSION

HDTs have gained increasing attention as a novel approach for TB due to their potential advantages in reducing treatment duration and dosage. HDTs achieve this by enhancing immune responses, making it less likely for Mtb strains to develop resistance. Moreover, certain HDT-based drugs have demonstrated significant antimicrobial efficacy against MDR isolates (140). Among the identified HDTs, strategies that boost phagosomal maturation and autophagy—well-explored in other diseases—are the most promising. Direct and indirect mTOR inhibitors, along with AMPK activators, stand out for their desirable clinical outcomes and the extensive libraries of relevant chemical compounds. Notably, metformin and everolimus have shown significant efficacy against Mtb in both *in vitro* and *in vivo* models through diverse HDT mechanisms. They have also exhibited synergistic or additive effects when combined with conventional antibiotics.

In contrast, most HDTs targeting phenotypic variation of macrophage and cell death regulation remain in the preclinical stage. These strategies, however, hold promise when used in combination to enhance therapeutic outcomes or mitigate adverse effects. For instance, autophagy-enhancement HDTs can be paired with those targeting phagosome maturation to counteract potential feedback effects. Furthermore, as more details about the interactions between macrophages and Mtb during TB pathogenesis are uncovered, and as novel technologies advance, the development of HDTs is poised to address the current challenges of TB treatment.

Despite their potential, the persistence of Mtb infection necessitates the combined use of HDT drugs with conventional antibiotics. This highlights the importance of exploring interactions between these therapies, yet only a limited number of studies have addressed this. Additionally, the diversity of patient populations, many of whom may have co-infections with other diseases, presents another challenge for immune response-modulating HDTs. This underscores the need for personalized HDT approaches, which should be considered in future research.

## PERSPECTIVE: FUTURE DIRECTIONS FOR HDTs IN TB MANAGEMENT

While HDTs offer a promising complement to traditional antibiotics in the fight against Mtb, their full potential remains untapped. Moving forward, several key areas warrant deeper exploration to address the current limitations and expand the applicability of HDTs:

### Unveiling mechanistic insights during TB

Taken together, most HDTs in macrophages target the mechanisms manipulated by Mtb, highlighting the critical importance of elucidating macrophage-Mtb interactions. However, many mechanistic details remain unclear, particularly the dynamic interplay between macrophage polarization and metabolic reprogramming during TB infection. Cutting-edge approaches, such as single-cell RNA sequencing and high-resolution imaging, enable a deeper dive into macrophage-Mtb interactions during TB infection, which offer unprecedented opportunities to identify novel and promising HDT targets.

### Personalized medicine approaches

As previously discussed, the sole inhibition of mTORC1 has been demonstrated to elevate Mtb burden in host cells co-infected with HIV, a finding that stands in stark contrast to the outcomes observed in HIV-uninfected host cells (32). Hence, the heterogeneity of patient populations, compounded by co-infections (e.g., HIV, diabetes) and varying immune profiles, potentially due to the suppression of phagosomal maturation caused by mTOR inhibitors, underscores the need for personalized HDTs. Integrating genomic, transcriptomic, and metabolomic data from diverse patient cohorts could guide the development of tailored HDTs, optimizing efficacy while minimizing adverse effects.

### Combination therapies and drug interactions with existing TB antibiotics

As HDTs are intended to complement existing TB antibiotics, understanding drug-drug interactions is critical, but only a limited number of these HDT drugs mentioned above have discussed this, where the marketed drugs among them are listed in Table 1. Studies exploring the synergistic effects of HDTs with standard regimens (e.g., RHZE) could refine combination therapy protocols. Furthermore, addressing potential antagonistic effects or unexpected immune suppression will ensure the safety and reliability of HDTs.

**TABLE 1:** Marketed drugs with HDT potential and anti-TB drug interactions^*a*^

Targets	Compounds	Combination	Refs.
Inhibition			
Sirtuin 5	Pimozide, fluspirilene	Synergistic and/or additive (R)	(67)
Abl tyrosine kinases	Imatinib	Synergistic (R)	(72, 73)
HMG-CoA	Simvastatin, fluvastatin, pravastatin	Synergistic (RHZE)	(77, 78)
mTOR	Rapamycin	No benefit (RHZE); Antagonism (BPal)	(30, 31, 36)
	Everolimus	Synergistic (H/Z)	(33–35, 37)
Poly (ADP-ribose) polymerase	Niraparib, pamiparib, rucaparib	Additive(R)	(95)
Histone deacetylase	Valproic acid, suberoylanilide hydroxamic acid	Additive (R/H)	(96)
Cholesterol absorption	Ezetimibe	Additive (R/H/Z); no effect with (E)	(114)
Phosphodiesterase-3	Cilostazol	Additive (R and RHZE)	(128)
Phosphodiesterase-5	Sildenafil	Additive (RHZE)	(128)
Activation			
AMPK	Metformin	Additive (H/E)	(45–47)
Transcript factor EB	Ambroxol	Additive (R)	(59)
	Tamoxifen	Synergistic (R)	(60, 62)
p38 MAPK	Biapenem	Synergistic (R)	(91)

^*a*^ R: rifampin, H: isoniazid, Z: pyrazinamide, E: ethambutol; AMPK: AMP-activated protein kinase; BPal: bedaquiline-pretomanid-linezolid; mTOR: the mammalian target of rapamycin.

### Optimizing drug delivery systems

Efficient delivery of HDT compounds to infected tissues, particularly granulomas, remains a challenge. Advances in nanotechnology, such as liposomal or nanoparticle-based drug delivery systems, could improve HDT bioavailability through tissue, cell, and even subcellular targeting (141). And this is particularly relevant for compounds like metformin and everolimus, where localized delivery might enhance therapeutic effects while minimizing systemic side effects.

By addressing these critical areas, HDTs could significantly reshape TB management, offering more effective, personalized, and sustainable treatment options. As we advance, integrating HDTs into comprehensive TB care frameworks has the potential to not only improve patient outcomes but also contribute to global efforts to eliminate TB as a public health threat.

## SUPPLEMENTAL MATERIAL

Table S1 iai.00063-25-s0001.docx HDTs targets involved in phagocytosis and autophagy of macrophages.

Table S2 iai.00063-25-s0002.docx HDT targets involved in metabolic changes during phenotypic variation of macrophages and cell death regulation in macrophages.

ASM does not own the copyrights to Supplemental Material that may be linked to, or accessed through, an article. The authors have granted ASM a non-exclusive, world-wide license to publish the Supplemental Material files. Please contact the corresponding author directly for reuse.
