# Supplementary material for: Host-directed therapeutic targets in macrophages and their ligands against mycobacteria tuberculosis
Source: Infect Immun. 2025 Aug 25;93(10):e00063-25. doi: 10.1128/iai.00063-25 (PMC12519779; doi:10.1128/iai.00063-25)
Supplement: Table S1 — HDTs targets involved in phagocytosis and autophagy of macrophages. [file iai.00063-25-s0001.docx]

Table 1 HDTs targets involved in phagocytosis and autophagy of macrophages

| Targets | | Compounds | Effect besides reducing CFU | | Combination | | Model | | *Pathogen* | | Toxicity | | Ref | |
| --- | --- | --- | --- | --- | --- | --- | --- | --- | --- | --- | --- | --- | --- | --- |
| Inhibition | |  | | | | | | | | | | | | |
| Adenylate cyclase  (1) | | SQ22536 | - | | - | | In vitro | | *HR37v* | | - | | 1 | |
| Protein kinase A  (1) | | H-89 | - | | - | | In vitro | | *HR37v* | | - | | 1 | |
|  |  | 97i | - | | - | | In vitro | | *HR37v* | | Slight | | 2 | |
| Sirtuin 5  (2) | | Pimozide, fluspirilene | - | | Synergistic and/or additive (R) | | In vitro | | *H37Rv* | | No | | 3 | |
| Src tyrosine kinases  (1, 2, 3) | | AZD0530 | Reduce necrotic granulomas | | - | | In vivo | | *H37Rv*, *JAL2261*, *1934*, *MYC431* | | High at high dose | | 4 | |
| Abl tyrosine kinases  (2) | | Imatinib | Reduce number of granulomatous lesions | | Synergistic (R) | | In vitro, in vivo | | *H37Rv, Erdman*  *M. marinum* | | Probable toxicity with long-term exposure | | 5,6 | |
| -  (2) | | Gefitinib | - | | - | | In vitro, in vivo | | *Erdman and H37Rv* | | - | | 7 | |
| HMG-CoA  (1, 3) | | Simvastatin | Reduce treatment duration and relapse rate | | Synergistic (RHZE) | | In vitro, in vivo, (R)CT | | *-* | | Slight | | 8,9 | |
|  |  | Fluvastatin, pravastatin | - | | Synergistic (RHZE) | | In vitro, in vivo (R)CT | | - | | Slight | | 8,9 | |
| mTOR  (3) | | Rapamycin | Reduce necrotic lesions and relapse rate | | No benefit (RHZE); Antagonism (BPal) | | In vivo | | *Erdman, H37Rv, HN878* | | - | | 10–12 | |
|  |  | CC214-2 | Reduce treatment duration and relapse rate | | Enhance with RHZE; No benefit with BPal | | In vivo | | *HN878* | | - | | 12 | |
|  |  | Everolimus | Reduce lipid droplets | | Synergistic (H/Z) | | In vitro, (R)CT | | *-* | | - | | 13–16 | |
| Akt/mTOR signaling pathway  (3) | | Baicalin | - | | - | | In vitro | | *H37Ra* | | Slight | |  | |
|  |  | Ibrutinib | - | | - | | In vitro, ex vivo | | *H37Ra, H37Rv* | | - | | 17 | |
|  |  | Bazedoxifene | - | | - | | In vitro | | *H37Ra, H37Rv* | | High | | 18 | |
| Activation | |  | | | | | | | | | | | | |
| Ca2+/CaM/CaMKII signaling pathway  (1) | | Flunarizine | - | | - | | In vitro | | *H37Ra, H37Rv* | | Slight | | 19,20 | |
|  |  | Verapamil, dantrolene, felodipine | - | | - | | In vitro | | *H37Rv* | | Slight | | 19,20 | |
| Sirtuin 1  (1, 3) | Resveratrol, SRT1720 | | | - | Enhance with H | In vitro, in vivo | | Multiple *Mtb* | | - | | 21 | |  |
| AMPK  (1, 3) | AICAR | | | - | - | In vitro, in vivo | | *H37Rv*  *M. bovis BCG*  *M. marinum* | | - | | 22 | |  |
|  | Carbamazepine | | | - | - | In vitro, in vivo | | *CSU 87* | | - | | 23 | |  |
|  | Metformin | | | a | Additive (H/E) | In vitro, in vivo, (R)CT | | *-* | | - | | 24–26 | |  |
| Type A GABA receptor  (1, 3) | GABA, muscimol, isoguvacine hydrochloride | | | - | - | In vitro, in vivo | | *H37Rv*  *M. bovis BCG* | | - | | 27 | |  |
| ERK1/2  (3) | Pasakbimin A | | | - | Enhance with R/H and RH | In vitro | | *H37Rv* | | Probably increased MMP-1/9 | | 28 | |  |
| P2RX7  (1, 3) | Vitamin D | | | b | - | In vitro, (R)CT | | *-* | | - | | 29–31 | |  |
|  | Phenylbutyrate | | | - | - | In vitro, (R)CT | | *-* | | - | | 30,32 | |  |
|  | Dehydroepian- drosterone | | | - | - | In vitro | | *H37Rv* | | No | | `33 | |  |
|  | Calcimycin | | | - | - | In vitro | | *M. smegmatis mc2155*  *M. bovis BCG* | | High at high dose | | 34 | |  |
| NR1D1  (3) | GSK4112 | | | - | - | In vitro | | *H37Rv* | | - | | 35 | |  |
| PPARα  (3) | GW7647 | | | - | - | In vivo | | *H37Rv*  *M.* *bovis* *BCG* | | - | | 36 | |  |
| Sirtuin 3  (3) | Honokiol | | | - | - | In vitro, in vivo | | *H37Rv*  *M.* *bovis* *BCG* | | - | | 37 | |  |
| PPM1A  (3) | SMIP-30 | | | - | - | In vitro, in vivo | | *H37Rv*, *mc^2^6206* | | Slight | | 38 | |  |
| Transcript factor EB  (3) | Ambroxol | | | - | Additive (R) | In vitro, in vivo | | *Erdman* | | - | | 39 | |  |
|  | Tamoxifen | | | - | Synergistic (R) | In vitro, in vivo | | *Multiple Mtb* | | High at high dose | | 40,41 | |  |

1: Promoting phagosomal maturation; 2: Promoting phagosomal acidification; 3: Promoting autophagy; R: rifampin, H: isoniazid, Z: pyrazinamide, E: ethambutol; AICAR: 5-aminoimidazole-4-carboxamide-1-β-D-ribofuranoside; AMPK: AMP-activated protein kinase; BPal: bedaquiline-pretomanid-linezolid; CFU: colony forming units; GABA: gamma-aminobutyric acid; *Mtb*: *Mycobacterium tuberculosis*; mTOR: the mammalian target of rapamycin; (R)CT: (randomized) clinical trial; a: Improve sputum culture conversion rate for patients with cavitary pulmonary TB and diabetes mellitus; b: Improve sputum culture conversion rate for patients.

**Reference**

(1) Kalamidas, S. A.; Kuehnel, M. P.; Peyron, P.; Rybin, V.; Rauch, S.; Kotoulas, O. B.; Houslay, M.; Hemmings, B. A.; Gutierrez, M. G.; Anes, E.; Griffiths, G. cAMP Synthesis and Degradation by Phagosomes Regulate Actin Assembly and Fusion Events: Consequences for Mycobacteria. *J. Cell Sci.* **2006**, *119* (17), 3686–3694. https://doi.org/10.1242/jcs.03091.

(2) Moreira, J. D.; Koch, B. E. V.; van Veen, S.; Walburg, K. V.; Vrieling, F.; Mara Pinto Dabés Guimarães, T.; Meijer, A. H.; Spaink, H. P.; Ottenhoff, T. H. M.; Haks, M. C.; Heemskerk, M. T. Functional Inhibition of Host Histone Deacetylases (HDACs) Enhances in Vitro and in Vivo Anti-Mycobacterial Activity in Human Macrophages and in Zebrafish. *Front. Immunol.* **2020**, *11*, 36. https://doi.org/10.3389/fimmu.2020.00036.

(3) Heemskerk, M. T.; Korbee, C. J.; Esselink, J. J.; dos Santos, C. C.; van Veen, S.; Gordijn, I. F.; Vrieling, F.; Walburg, K. V.; Engele, C. G.; Dijkman, K.; Wilson, L.; Verreck, F. a. W.; Ottenhoff, T. H. M.; Haks, M. C. Repurposing Diphenylbutylpiperidine-Class Antipsychotic Drugs for Host-Directed Therapy of Mycobacterium Tuberculosis and Salmonella Enterica Infections. *Sci. Rep.* **2021**, *11* (1), 1–18. https://doi.org/10.1038/s41598-021-98980-z.

(4) Chandra, P.; Rajmani, R. S.; Verma, G.; Bhavesh, N. S.; Kumar, D. Targeting Drug-Sensitive and -Resistant Strains of Mycobacterium Tuberculosis by Inhibition of Src Family Kinases Lowers Disease Burden and Pathology. *mSphere* **2016**, *1* (2), e00043-15. https://doi.org/10.1128/mSphere.00043-15.

(5) Napier, R. J.; Rafi, W.; Cheruvu, M.; Powell, K. R.; Zaunbrecher, M. A.; Bornmann, W.; Salgame, P.; Shinnick, T. M.; Kalman, D. Imatinib-Sensitive Tyrosine Kinases Regulate Mycobacterial Pathogenesis and Represent Therapeutic Targets against Tuberculosis. *Cell Host Microbe* **2011**, *10* (5), 475–485. https://doi.org/10.1016/j.chom.2011.09.010.

(6) Bruns, H.; Stegelmann, F.; Fabri, M.; Döhner, K.; van Zandbergen, G.; Wagner, M.; Skinner, M.; Modlin, R. L.; Stenger, S. Abelson Tyrosine Kinase Controls Phagosomal Acidification Required for Killing of *Mycobacterium Tuberculosis* in Human Macrophages. *J. Immunol.* **2012**, *189* (8), 4069–4078. https://doi.org/10.4049/jimmunol.1201538.

(7) Sogi, K. M.; Lien, K. A.; Johnson, J. R.; Krogan, N. J.; Stanley, S. A. The Tyrosine Kinase Inhibitor Gefitinib Restricts Mycobacterium Tuberculosis Growth through Increased Lysosomal Biogenesis and Modulation of Cytokine Signaling. *ACS Infect. Dis.* **2017**, *3* (8), 564–574. https://doi.org/10.1021/acsinfecdis.7b00046.

(8) Parihar, S. P.; Guler, R.; Khutlang, R.; Lang, D. M.; Hurdayal, R.; Mhlanga, M. M.; Suzuki, H.; Marais, A. D.; Brombacher, F. Statin Therapy Reduces the Mycobacterium Tuberculosis Burden in Human Macrophages and in Mice by Enhancing Autophagy and Phagosome Maturation. *J. Infect. Dis.* **2014**, *209* (5), 754–763. https://doi.org/10.1093/infdis/jit550.

(9) Dutta, N. K.; Bruiners, N.; Zimmerman, M. D.; Tan, S.; Dartois, V.; Gennaro, M. L.; Karakousis, P. C. Adjunctive Host-Directed Therapy With Statins Improves Tuberculosis-Related Outcomes in Mice. *J. Infect. Dis.* **2020**, *221* (7), 1079–1087. https://doi.org/10.1093/infdis/jiz517.

(10) Bhatt, K.; Bhagavathula, M.; Verma, S.; Timmins, G. S.; Deretic, V. P.; Ellner, J. J.; Salgame, P. Rapamycin Modulates Pulmonary Pathology in a Murine Model of Mycobacterium Tuberculosis Infection. *Dis. Model. Mech.* **2021**, *14* (10), dmm049018. https://doi.org/10.1242/dmm.049018.

(11) Gupta, A.; Sharma, D.; Meena, J.; Pandya, S.; Sachan, M.; Kumar, S.; Singh, K.; Mitra, K.; Sharma, S.; Panda, A. K.; Gupta, P.; Gupta, U. D.; Misra, A. Preparation and Preclinical Evaluation of Inhalable Particles Containing Rapamycin and Anti-Tuberculosis Agents for Induction of Autophagy. *Pharm. Res.* **2016**, *33* (8), 1899–1912. https://doi.org/10.1007/s11095-016-1926-0.

(12) Tasneen, R.; Mortensen, D. S.; Converse, P. J.; Urbanowski, M. E.; Upton, A.; Fotouhi, N.; Nuermberger, E.; Hawryluk, N. Dual mTORC1/mTORC2 Inhibition as a Host-Directed Therapeutic Target in Pathologically Distinct Mouse Models of Tuberculosis. *Antimicrob. Agents Chemother.* **2021**, *65* (7), e00253-21. https://doi.org/10.1128/AAC.00253-21.

(13) Wallis, R. S.; Ginindza, S.; Beattie, T.; Arjun, N.; Likoti, M.; Edward, V. A.; Rassool, M.; Ahmed, K.; Fielding, K.; Ahidjo, B. A.; Vangu, M. D. T.; Churchyard, G. Adjunctive Host-Directed Therapies for Pulmonary Tuberculosis: A Prospective, Open-Label, Phase 2, Randomised Controlled Trial. *Lancet Respir. Med.* **2021**, *9* (8), 897–908. https://doi.org/10.1016/S2213-2600(20)30448-3.

(14) Ashley, D.; Hernandez, J.; Cao, R.; To, K.; Yegiazaryan, A.; Abrahem, R.; Nguyen, T.; Owens, J.; Lambros, M.; Subbian, S.; Venketaraman, V. Antimycobacterial Effects of Everolimus in a Human Granuloma Model. *J. Clin. Med.* **2020**, *9* (7), 2043. https://doi.org/10.3390/jcm9072043.

(15) Cerni, S.; Shafer, D.; To, K.; Venketaraman, V. Investigating the Role of Everolimus in mTOR Inhibition and Autophagy Promotion as a Potential Host-Directed Therapeutic Target in Mycobacterium Tuberculosis Infection. *J. Clin. Med.* **2019**, *8* (2), 232. https://doi.org/10.3390/jcm8020232.

(16) Cao, R.; To, K.; Kachour, N.; Beever, A.; Owens, J.; Sathananthan, A.; Singh, P.; Kolloli, A.; Subbian, S.; Venketaraman, V. Everolimus-Induced Effector Mechanism in Macrophages and Survivability of Erdman, CDC1551 and HN878 Strains of *Mycobacterium Tuberculosis* Infection. *Biomol. Concepts* **2021**, *12* (1), 46–54. https://doi.org/10.1515/bmc-2021-0006.

(17) Hu, Y.; Wen, Z.; Liu, S.; Cai, Y.; Guo, J.; Xu, Y.; Lin, D.; Zhu, J.; Li, D.; Chen, X. Ibrutinib Suppresses Intracellular Mycobacterium Tuberculosis Growth by Inducing Macrophage Autophagy. *J. Infect.* **2020**, *80* (6), e19–e26. https://doi.org/10.1016/j.jinf.2020.03.003.

(18) Ouyang, Q.; Zhang, K.; Lin, D.; Feng, C. G.; Cai, Y.; Chen, X. Bazedoxifene Suppresses Intracellular Mycobacterium Tuberculosis Growth by Enhancing Autophagy. *mSphere* **2020**, *5* (2), e00124-20. https://doi.org/10.1128/mSphere.00124-20.

(19) Meena, P. R.; Monu; Meena, L. S. Fibronectin Binding Protein and Ca ^2+^ Play an Access Key Role to Mediate Pathogenesis in *Mycobacterium Tuberculosis* : An Overview: Role of FnBP and Ca ^2+^ in Pathogenesis. *Biotechnol. Appl. Biochem.* **2016**, *63* (6), 820–826. https://doi.org/10.1002/bab.1434.

(20) Mo, S.; Liu, X.; Zhang, K.; Wang, W.; Cai, Y.; Ouyang, Q.; Zhu, C.; Lin, D.; Wan, H.; Li, D.; Wen, Z.; Chen, X. Flunarizine Suppresses *Mycobacterium Tuberculosis* Growth via Calmodulin‐dependent Phagosome Maturation. *J. Leukoc. Biol.* **2022**, *111* (5), 1021–1029. https://doi.org/10.1002/JLB.4A0221-119RR.

(21) Cheng, C. Y.; Gutierrez, N. M.; Marzuki, M. B.; Lu, X.; Foreman, T. W.; Paleja, B.; Lee, B.; Balachander, A.; Chen, J.; Tsenova, L.; Kurepina, N.; Teng, K. W. W.; West, K.; Mehra, S.; Zolezzi, F.; Poidinger, M.; Kreiswirth, B.; Kaushal, D.; Kornfeld, H.; Newell, E. W.; Singhal, A. Host Sirtuin 1 Regulates Mycobacterial Immunopathogenesis and Represents a Therapeutic Target against Tuberculosis. *Sci. Immunol.* **2017**, *2* (9), eaaj1789. https://doi.org/10.1126/sciimmunol.aaj1789.

(22) Yang, C.-S.; Kim, J.-J.; Lee, H.-M.; Jin, H. S.; Lee, S.-H.; Park, J.-H.; Kim, S. J.; Kim, J.-M.; Han, Y.-M.; Lee, M.-S.; Kweon, G. R.; Shong, M.; Jo, E.-K. The AMPK-PPARGC1A Pathway Is Required for Antimicrobial Host Defense through Activation of Autophagy. *Autophagy* **2014**, *10* (5), 785–802. https://doi.org/10.4161/auto.28072.

(23) Schiebler, M.; Brown, K.; Hegyi, K.; Newton, S. M.; Renna, M.; Hepburn, L.; Klapholz, C.; Coulter, S.; Obregón‐Henao, A.; Henao Tamayo, M.; Basaraba, R.; Kampmann, B.; Henry, K. M.; Burgon, J.; Renshaw, S. A.; Fleming, A.; Kay, R. R.; Anderson, K. E.; Hawkins, P. T.; Ordway, D. J.; Rubinsztein, D. C.; Floto, R. A. Functional Drug Screening Reveals Anticonvulsants as Enhancers of mTOR‐independent Autophagic Killing of *Mycobacterium Tuberculosis* through Inositol Depletion. *EMBO Mol. Med.* **2015**, *7* (2), 127–139. https://doi.org/10.15252/emmm.201404137.

(24) Padmapriydarsini, C.; Mamulwar, M.; Mohan, A.; Shanmugam, P.; Gomathy, N. S.; Mane, A.; Singh, U. B.; Pavankumar, N.; Kadam, A.; Kumar, H.; Suresh, C.; Reddy, D.; Devi, P.; Ramesh, P. M.; Sekar, L.; Jawahar, S.; Shandil, R. K.; Singh, M.; Menon, J.; Guleria, R.; the METRIF Team. Randomized Trial of Metformin With Anti-Tuberculosis Drugs for Early Sputum Conversion in Adults With Pulmonary Tuberculosis. *Clin. Infect. Dis.* **2022**, *75* (3), 425–434. https://doi.org/10.1093/cid/ciab964.

(25) Singhal, A.; Jie, L.; Kumar, P.; Hong, G. S.; Leow, M. K.-S.; Paleja, B.; Tsenova, L.; Kurepina, N.; Chen, J.; Zolezzi, F.; Kreiswirth, B.; Poidinger, M.; Chee, C.; Kaplan, G.; Wang, Y. T.; De Libero, G. Metformin as Adjunct Antituberculosis Therapy. *Sci. Transl. Med.* **2014**, *6* (263), 263ra159. https://doi.org/10.1126/scitranslmed.3009885.

(26) Lee, Y.-J.; Han, S. K.; Park, J. H.; Lee, J. K.; Kim, D. K.; Chung, H. S.; Heo, E. Y. The Effect of Metformin on Culture Conversion in Tuberculosis Patients with Diabetes Mellitus. *Korean J. Intern. Med.* **2018**, *33* (5), 933–940. https://doi.org/10.3904/kjim.2017.249.

(27) Kim, J. K.; Kim, Y. S.; Lee, H.-M.; Jin, H. S.; Neupane, C.; Kim, S.; Lee, S.-H.; Min, J.-J.; Sasai, M.; Jeong, J.-H.; Choe, S.-K.; Kim, J.-M.; Yamamoto, M.; Choy, H. E.; Park, J. B.; Jo, E.-K. GABAergic Signaling Linked to Autophagy Enhances Host Protection against Intracellular Bacterial Infections. *Nat. Commun.* **2018**, *9* (1), 4184. https://doi.org/10.1038/s41467-018-06487-5.

(28) Lee, H.-J.; Ko, H.-J.; Kim, S. H.; Jung, Y.-J. Pasakbumin A Controls the Growth of Mycobacterium Tuberculosis by Enhancing the Autophagy and Production of Antibacterial Mediators in Mouse Macrophages. *PLOS ONE* **2019**, *14* (3), e0199799. https://doi.org/10.1371/journal.pone.0199799.

(29) Liu, P. T.; Stenger, S.; Tang, D. H.; Modlin, R. L. Cutting Edge: Vitamin D-Mediated Human Antimicrobial Activity against *Mycobacterium Tuberculosis* Is Dependent on the Induction of Cathelicidin. *J. Immunol.* **2007**, *179* (4), 2060–2063. https://doi.org/10.4049/jimmunol.179.4.2060.

(30) Bekele, A.; Gebreselassie, N.; Ashenafi, S.; Kassa, E.; Aseffa, G.; Amogne, W.; Getachew, M.; Aseffa, A.; Worku, A.; Raqib, R.; Agerberth, B.; Hammar, U.; Bergman, P.; Aderaye, G.; Andersson, J.; Brighenti, S. Daily Adjunctive Therapy with Vitamin D3 and Phenylbutyrate Supports Clinical Recovery from Pulmonary Tuberculosis: A Randomized Controlled Trial in Ethiopia. *J. Intern. Med.* **2018**, *284* (3), 292–306. https://doi.org/10.1111/joim.12767.

(31) Martineau, A. R.; Timms, P. M.; Bothamley, G. H.; Hanifa, Y.; Islam, K.; Claxton, A. P.; Packe, G. E.; Moore-Gillon, J. C.; Darmalingam, M.; Davidson, R. N.; Milburn, H. J.; Baker, L. V.; Barker, R. D.; Woodward, N. J.; Venton, T. R.; Barnes, K. E.; Mullett, C. J.; Coussens, A. K.; Rutterford, C. M.; Mein, C. A.; Davies, G. R.; Wilkinson, R. J.; Nikolayevskyy, V.; Drobniewski, F. A.; Eldridge, S. M.; Griffiths, C. J. High-Dose Vitamin D3 during Intensive-Phase Antimicrobial Treatment of Pulmonary Tuberculosis: A Double-Blind Randomised Controlled Trial. *The Lancet* **2011**, *377* (9761), 242–250. https://doi.org/10.1016/S0140-6736(10)61889-2.

(32) Rekha, R. S.; Rao Muvva, S. J.; Wan, M.; Raqib, R.; Bergman, P.; Brighenti, S.; Gudmundsson, G. H.; Agerberth, B. Phenylbutyrate Induces LL-37-Dependent Autophagy and Intracellular Killing of *Mycobacterium Tuberculosis* in Human Macrophages. *Autophagy* **2015**, *11* (9), 1688–1699. https://doi.org/10.1080/15548627.2015.1075110.

(33) Marin-Luevano, S. P.; Rodriguez-Carlos, A.; Jacobo-Delgado, Y.; Valdez-Miramontes, C.; Enciso-Moreno, J. A.; Rivas-Santiago, B. Steroid Hormone Modulates the Production of Cathelicidin and Human β-Defensins in Lung Epithelial Cells and Macrophages Promoting Mycobacterium Tuberculosis Killing. *Tuberculosis* **2021**, *128*, 102080. https://doi.org/10.1016/j.tube.2021.102080.

(34) Mawatwal, S.; Behura, A.; Ghosh, A.; Kidwai, S.; Mishra, A.; Deep, A.; Agarwal, S.; Saha, S.; Singh, R.; Dhiman, R. Calcimycin Mediates Mycobacterial Killing by Inducing Intracellular Calcium-Regulated Autophagy in a P2RX7 Dependent Manner. *Biochim. Biophys. Acta BBA - Gen. Subj.* **2017**, *1861* (12), 3190–3200. https://doi.org/10.1016/j.bbagen.2017.09.010.

(35) Chandra, V.; Bhagyaraj, E.; Nanduri, R.; Ahuja, N.; Gupta, P. NR1D1 Ameliorates *Mycobacterium Tuberculosis* Clearance through Regulation of Autophagy. *Autophagy* **2015**, *11* (11), 1987–1997. https://doi.org/10.1080/15548627.2015.1091140.

(36) Kim, Y. S.; Lee, H.-M.; Kim, J. K.; Yang, C.-S.; Kim, T. S.; Jung, M.; Jin, H. S.; Kim, S.; Jang, J.; Oh, G. T.; Kim, J.-M.; Jo, E.-K. PPAR-α Activation Mediates Innate Host Defense through Induction of TFEB and Lipid Catabolism. *J. Immunol.* **2017**, *198* (8), 3283–3295. https://doi.org/10.4049/jimmunol.1601920.

(37) Kim, T. S.; Jin, Y. B.; Kim, Y. S.; Kim, S.; Kim, J. K.; Lee, H.-M.; Suh, H.-W.; Choe, J. H.; Kim, Y. J.; Koo, B.-S.; Kim, H.-N.; Jung, M.; Lee, S.-H.; Kim, D.-K.; Chung, C.; Son, J.-W.; Min, J.-J.; Kim, J.-M.; Deng, C.-X.; Kim, H. S.; Lee, S.-R.; Jo, E.-K. SIRT3 Promotes Antimycobacterial Defenses by Coordinating Mitochondrial and Autophagic Functions. *Autophagy* **2019**, *15* (8), 1356–1375. https://doi.org/10.1080/15548627.2019.1582743.

(38) Berton, S.; Chen, L.; Liang, Y. C.; Xu, Z.; Afriyie-Asante, A.; Rajabalee, N.; Yang, W.; Sun, J. A Selective PPM1A Inhibitor Activates Autophagy to Restrict the Survival of Mycobacterium Tuberculosis. *Cell Chem. Biol.* **2022**, *29* (7), 1126-1139.e12. https://doi.org/10.1016/j.chembiol.2022.03.006.

(39) Choi, S. W.; Gu, Y.; Peters, R. S.; Salgame, P.; Ellner, J. J.; Timmins, G. S.; Deretic, V. Ambroxol Induces Autophagy and Potentiates Rifampin Antimycobacterial Activity. *Antimicrob. Agents Chemother.* **2018**, *62* (9), e01019-18. https://doi.org/10.1128/AAC.01019-18.

(40) Boland, R.; Heemskerk, M. T.; Forn-Cuní, G.; Korbee, C. J.; Walburg, K. V.; Esselink, J. J.; Carvalho Dos Santos, C.; De Waal, A. M.; Van Der Hoeven, D. C. M.; Van Der Sar, E.; De Ries, A. S.; Xie, J.; Spaink, H. P.; Van Der Vaart, M.; Haks, M. C.; Meijer, A. H.; Ottenhoff, T. H. M. Repurposing Tamoxifen as Potential Host-Directed Therapeutic for Tuberculosis. *mBio* **2023**, *14* (1), e03024-22. https://doi.org/10.1128/mbio.03024-22.

(41) Jang, W. S.; Kim, S.; Podder, B.; Jyoti, M. A.; Nam, K.-W.; Song, B.-E. L. and H.-Y. Anti-Mycobacterial Activity of Tamoxifen Against Drug-Resistant and Intra-Macrophage Mycobacterium Tuberculosis. **2015**, *25* (6), 946–950. https://doi.org/10.4014/jmb.1412.12023.
