# Supplementary material for: Host-directed therapeutic targets in macrophages and their ligands against mycobacteria tuberculosis
Source: Infect Immun. 2025 Aug 25;93(10):e00063-25. doi: 10.1128/iai.00063-25 (PMC12519779; doi:10.1128/iai.00063-25)
Supplement: Table S2 — HDT targets involved in metabolic changes during phenotypic variation of macrophages and cell death regulation in macrophages. [file iai.00063-25-s0002.docx]

Table 2 HDT targets involved in metabolic changes during phenotypic variation of macrophages and cell death regulation in macrophages

| **Targets** | **Compounds** | **Mechanisms of reducing bacterial load** | **Combination** | **Model** | **Pathogen** | **Toxicity** | **Ref** |
| --- | --- | --- | --- | --- | --- | --- | --- |
| **Inhibition** |  | | | | | | |
| PI3K | Wortmannin | M1 polarization↑ | - | In vitro | *M.* *bovis* *BCG* | High at high dose | 1 |
| Akt | MK-2206 | M1 polarization↑ | - | In vitro | *M.* *bovis* *BCG* | High at high lose | 1 |
| Poly (ADP-ribose) polymerase | Niraparib, pamiparib, rucaparib, A-966492 | Antimicrobial ability↑ | Additive(R) | In vitro | Multiple *Mtb*  *M.* avium | No | 2 |
| Histone deacetylase | Valproic acid, SAHA | Antimicrobial ability↑ | Additive (R/H) | In vitro | *H37Rv* | - | 3 |
|  | Phenylbutyrate | Antimicrobial ability↑ | - | In vitro, (R)CT | - | - | 4,5 |
|  | Trichostatin A, TMP195, TMP269 | Antimicrobial ability↑ | - | In vitro, in vivo | *H37Rv*  *M.* *marinum* | No | 6 |
| xCT | Sulfasalazine, (S)-4-carboxy- phenylglycine | Oxidative stress↑ | - | In vivo | *H37Rv*, *H37Ra* | No | 7 |
| Heme oxygenase-1 | Tin protoporphyrin | Probably ROS↑ | Additive (RHZ) | In vivo, in vitro | *H37Rv*,  *Erdman* | - | 8 |
| NAD(P)H dehydrogenase, quinone 1 | Dicoumarol | Probably ROS↑ | Synergistic (R) | In vitro | *H37Rv,*  *M. bovis BCG* | No | 9 |
|  | ES936 | Probably ROS↑ | - | In vitro | *H37Rv,*  *M. bovis BCG* | No | 9 |
|  | Nitazoxanide, tizoxanide | Probably ROS↑ | - | In vitro | *H37Rv,*  *M. bovis BCG* | High at high lose | 9 |
| Fatty acids oxidation | Etomoxir, timetazidine, oxfenicine | ROS↑ | - | In vitro, in vivo | *H37Rv* | No | 10,11 |
| Cholesterol absorption | Ezetimibe | Lipid accumulation↓ | Additive (R/H/Z); No effect with (E) | In vitro | *-* | - | 12 |
| Diacylglycerol-O- acyltransferases 1 | T863 | Lipid accumulation↓ | - | In vivo | *Erdman* | - | 13 |
| GPR109A | Mepenzolate bromide | Lipid accumulation↓ | - | In vitro, in vivo | Multiple *Mtb* | No | 14 |
| Mcl-1 | Sabutoclax, TW-37 | Apoptosis↑ | - | In vitro | *H37Rv* | Slight | 15 |
| 15-lipoxygenase | PD146176 | Apoptosis↑ | - | In vitro | *H37Rv* | - | 15 |
| Inhibitor of apoptosis proteins | LCL161 | Apoptosis↑ | - | In vivo | *H37Rv* | - | 16 |
| Sirtuin 2 | AGK2 | Apoptosis↑  Necrosis↓ | - | Ex vivo, in vivo | Multiple *Mtb* | - | 17 |
| Phosphodiesterase-3 | Cilostazol | Necroptosis↓ | Additive (R and RHZE) | In vivo | *H37Rv, CDC1551* | No | 18 |
| Phosphodiesterase-5 | Sildenafil | Necroptosis↓ | Additive (RHZE) | In vivo | *H37Rv, CDC1551* | No | 18 |
| Cyclophilin D | Alisporivir | Necroptosis↓ | - | In vivo | *M.* *marinum* | Moderate | 19 |
| Acid sphingomyelinase | Desipramine | Necroptosis↓ | - | In vivo | *M.* *marinum* | Moderate | 19 |
| Lipid peroxidation | Ferrostatin 1 | Ferroptosis↓ | - | In vitro, in vivo | *H37Rv* | - | 20 |
| **Activation** |  | | | | | | |
| p38 MAPK | Biapenem | M1 polarization↑ | Synergistic (R) | In vitro, ex vivo, in vivo | *H37Rv, Rv-GFP*  *JAL 2261,*  *MYC 431* | - | 21 |
|  | SQ109 | M1 polarization↑ | Enhance with H | In vitro, in vivo, ex vivo | *H37Rv*, *Jal2261*, *MYC431* | - | 22 |
|  | Rocaglates like CMLD010536 | M1 polarization↑ | - | In vitro | *H37Rv* | No | 23 |
|  | Withaferin A | M1 polarization↑ | Synergistic (H) | In vitro, in vivo, ex vivo | *H37Rv, Rv-GFP, JAL2261, MYC431* | No | 24 |
| HIF-1α | CoCl2 | Glycolysis↑ | Enhance with R | In vitro | *H37Rv* | - | 25 |
| JNK pathway | Anisomycin | Apoptosis↑ | Additive (R) | In vitro | *mc^2^6206* | No | 26 |

*Mtb*: *Mycobacterium tuberculosis*; R: rifampin, H: isoniazid, Z: pyrazinamide, E: ethambutol; ROS: reactive oxygen species; SAHA: suberoylanilide hydroxamic acid.

**Reference**

(1) Bouzeyen, R.; Haoues, M.; Barbouche, M.-R.; Singh, R.; Essafi, M. FOXO3 Transcription Factor Regulates IL-10 Expression in Mycobacteria-Infected Macrophages, Tuning Their Polarization and the Subsequent Adaptive Immune Response. *Front. Immunol.* **2019**, *10*. https://doi.org/10.3389/fimmu.2019.02922.

(2) van Doorn, C. L. R.; Steenbergen, S. A. M.; Walburg, K. V.; Ottenhoff, T. H. M. Pharmacological Poly (ADP-Ribose) Polymerase Inhibitors Decrease Mycobacterium Tuberculosis Survival in Human Macrophages. *Front. Immunol.* **2021**, *0*. https://doi.org/10.3389/fimmu.2021.712021.

(3) Rao, M.; Valentini, D.; Zumla, A.; Maeurer, M. Evaluation of the Efficacy of Valproic Acid and Suberoylanilide Hydroxamic Acid (Vorinostat) in Enhancing the Effects of First-Line Tuberculosis Drugs against Intracellular Mycobacterium Tuberculosis. *International Journal of Infectious Diseases* **2018**, *69*, 78–84. https://doi.org/10.1016/j.ijid.2018.02.021.

(4) Rekha, R. S.; Rao Muvva, S. J.; Wan, M.; Raqib, R.; Bergman, P.; Brighenti, S.; Gudmundsson, G. H.; Agerberth, B. Phenylbutyrate Induces LL-37-Dependent Autophagy and Intracellular Killing of *Mycobacterium Tuberculosis* in Human Macrophages. *Autophagy* **2015**, *11* (9), 1688–1699. https://doi.org/10.1080/15548627.2015.1075110.

(5) Bekele, A.; Gebreselassie, N.; Ashenafi, S.; Kassa, E.; Aseffa, G.; Amogne, W.; Getachew, M.; Aseffa, A.; Worku, A.; Raqib, R.; Agerberth, B.; Hammar, U.; Bergman, P.; Aderaye, G.; Andersson, J.; Brighenti, S. Daily Adjunctive Therapy with Vitamin D3 and Phenylbutyrate Supports Clinical Recovery from Pulmonary Tuberculosis: A Randomized Controlled Trial in Ethiopia. *J Intern Med* **2018**, *284* (3), 292–306. https://doi.org/10.1111/joim.12767.

(6) Moreira, J. D.; Koch, B. E. V.; van Veen, S.; Walburg, K. V.; Vrieling, F.; Mara Pinto Dabés Guimarães, T.; Meijer, A. H.; Spaink, H. P.; Ottenhoff, T. H. M.; Haks, M. C.; Heemskerk, M. T. Functional Inhibition of Host Histone Deacetylases (HDACs) Enhances in Vitro and in Vivo Anti-Mycobacterial Activity in Human Macrophages and in Zebrafish. *Front Immunol* **2020**, *11*, 36. https://doi.org/10.3389/fimmu.2020.00036.

(7) Cai, Y.; Yang, Q.; Liao, M.; Wang, H.; Zhang, C.; Nambi, S.; Wang, W.; Zhang, M.; Wu, J.; Deng, G.; Deng, Q.; Liu, H.; Zhou, B.; Jin, Q.; Feng, C. G.; Sassetti, C. M.; Wang, F.; Chen, X. xCT Increases Tuberculosis Susceptibility by Regulating Antimicrobial Function and Inflammation. *Oncotarget* **2016**, *7* (21), 31001–31013. https://doi.org/10.18632/oncotarget.9052.

(8) Scharn, C. R.; Collins, A. C.; Nair, V. R.; Stamm, C. E.; Marciano, D. K.; Graviss, E. A.; Shiloh, M. U. Heme Oxygenase-1 Regulates Inflammation and Mycobacterial Survival in Human Macrophages during Mycobacterium Tuberculosis Infection. *The Journal of Immunology* **2016**, *196* (11), 4641–4649. https://doi.org/10.4049/jimmunol.1500434.

(9) Li, Q.; Karim, A. F.; Ding, X.; Das, B.; Dobrowolski, C.; Gibson, R. M.; Quiñones-Mateu, M. E.; Karn, J.; Rojas, R. E. Novel High Throughput Pooled shRNA Screening Identifies NQO1 as a Potential Drug Target for Host Directed Therapy for Tuberculosis. *Sci Rep* **2016**, *6* (1), 27566. https://doi.org/10.1038/srep27566.

(10) Huang, L.; Nazarova, E. V.; Tan, S.; Liu, Y.; Russell, D. G. Growth of Mycobacterium Tuberculosis in Vivo Segregates with Host Macrophage Metabolism and Ontogeny. *J Exp Med* **2018**, *215* (4), 1135–1152. https://doi.org/10.1084/jem.20172020.

(11) Chandra, P.; He, L.; Zimmerman, M.; Yang, G.; Köster, S.; Ouimet, M.; Wang, H.; Moore, K. J.; Dartois, V.; Schilling, J. D.; Philips, J. A. Inhibition of Fatty Acid Oxidation Promotes Macrophage Control of Mycobacterium Tuberculosis. *Mbio* **2020**, *11* (4), e01139-20. https://doi.org/10.1128/mBio.01139-20.

(12) Tsai, I.-F.; Kuo, C.-P.; Lin, A. B.; Chien, M.-N.; Ho, H.-T.; Wei, T.-Y.; Wu, C.-L.; Lu, Y.-T. Potential Effect of Ezetimibe against *Mycobacterium Tuberculosis* Infection in Type II Diabetes: Ezetimibe Effect on TB. *Respirology* **2017**, *22* (3), 559–566. https://doi.org/10.1111/resp.12948.

(13) Dawa, S.; Menon, D.; Arumugam, P.; Bhaskar, A. K.; Mondal, M.; Rao, V.; Gandotra, S. Inhibition of Granuloma Triglyceride Synthesis Imparts Control of Mycobacterium Tuberculosis Through Curtailed Inflammatory Responses. *Front. Immunol.* **2021**, *0*. https://doi.org/10.3389/fimmu.2021.722735.

(14) Singh, V.; Jamwal, S.; Jain, R.; Verma, P.; Gokhale, R.; Rao, K. V. S. Mycobacterium Tuberculosis-Driven Targeted Recalibration of Macrophage Lipid Homeostasis Promotes the Foamy Phenotype. *Cell Host Microbe* **2012**, *12* (5), 669–681. https://doi.org/10.1016/j.chom.2012.09.012.

(15) Arnett, E.; Weaver, A. M.; Woodyard, K. C.; Montoya, M. J.; Li, M.; Hoang, K. V.; Hayhurst, A.; Azad, A. K.; Schlesinger, L. S. PPARγ Is Critical for Mycobacterium Tuberculosis Induction of Mcl-1 and Limitation of Human Macrophage Apoptosis. *Plos Pathog* **2018**, *14* (6), e1007100. https://doi.org/10.1371/journal.ppat.1007100.

(16) Stutz, M. D.; Allison, C. C.; Ojaimi, S.; Preston, S. P.; Doerflinger, M.; Arandjelovic, P.; Whitehead, L.; Bader, S. M.; Batey, D.; Asselin-Labat, M.-L.; Herold, M. J.; Strasser, A.; West, N. P.; Pellegrini, M. Macrophage and Neutrophil Death Programs Differentially Confer Resistance to Tuberculosis. *Immunity* **2021**, *54* (8), 1758-1771.e7. https://doi.org/10.1016/j.immuni.2021.06.009.

(17) Bhaskar, A.; Kumar, S.; Khan, M. Z.; Singh, A.; Dwivedi, V. P.; Nandicoori, V. K. Host Sirtuin 2 as an Immunotherapeutic Target against Tuberculosis. *eLife* **2020**, *9*, e55415. https://doi.org/10.7554/eLife.55415.

(18) Maiga, M.; Agarwal, N.; Ammerman, N. C.; Gupta, R.; Guo, H.; Maiga, M. C.; Lun, S.; Bishai, W. R. Successful Shortening of Tuberculosis Treatment Using Adjuvant Host-Directed Therapy with FDA-Approved Phosphodiesterase Inhibitors in the Mouse Model. *PLoS ONE* **2012**, *7* (2), e30749. https://doi.org/10.1371/journal.pone.0030749.

(19) Roca, F. J.; Ramakrishnan, L. TNF Dually Mediates Resistance and Susceptibility to Mycobacteria via Mitochondrial Reactive Oxygen Species. *Cell* **2013**, *153* (3), 521–534. https://doi.org/10.1016/j.cell.2013.03.022.

(20) Amaral, E. P.; Costa, D. L.; Namasivayam, S.; Riteau, N.; Kamenyeva, O.; Mittereder, L.; Mayer-Barber, K. D.; Andrade, B. B.; Sher, A. A Major Role for Ferroptosis in Mycobacterium Tuberculosis–Induced Cell Death and Tissue Necrosis. *J Exp Med* **2019**, *216* (3), 556–570. https://doi.org/10.1084/jem.20181776.

(21) Pahuja, I.; Verma, A.; Ghoshal, A.; Mukhopadhyay, S.; Kumari, A.; Shaji, A.; Chaturvedi, S.; Dwivedi, V. P.; Bhaskar, A. Biapenem, a Carbapenem Antibiotic, Elicits Mycobacteria Specific Immune Responses and Reduces the Recurrence of Tuberculosis. *Microbiology Spectrum* **2023**, *0* (0), e00858-23. https://doi.org/10.1128/spectrum.00858-23.

(22) Singh, M.; Kumar, S.; Singh, B.; Jain, P.; Kumari, A.; Pahuja, I.; Chaturvedi, S.; Prasad, D. V. R.; Dwivedi, V. P.; Das, G. The 1, 2-Ethylenediamine SQ109 Protects against Tuberculosis by Promoting M1 Macrophage Polarization through the P38 MAPK Pathway. *Commun Biol* **2022**, *5* (1), 759. https://doi.org/10.1038/s42003-022-03693-2.

(23) Chatterjee, S.; Yabaji, S. M.; Rukhlenko, O. S.; Bhattacharya, B.; Waligurski, E.; Vallavoju, N.; Ray, S.; Kholodenko, B. N.; Brown, L. E.; Beeler, A. B.; Ivanov, A. R.; Kobzik, L.; Porco, J. A.; Kramnik, I. Channeling Macrophage Polarization by Rocaglates Increases Macrophage Resistance to Mycobacterium Tuberculosis. *Iscience* **2021**, *24* (8), 102845. https://doi.org/10.1016/j.isci.2021.102845.

(24) Kumari, A.; Pahuja, I.; Negi, K.; Ghoshal, A.; Mukopadhyay, S.; Agarwal, M.; Mathew, B.; Maras, J. S.; Chaturvedi, S.; Bhaskar, A.; Dwivedi, V. P. Withaferin A Protects against Primary and Recurrent Tuberculosis by Modulating Mycobacterium-Specific Host Immune Responses. *Microbiol Spectr* *11* (2), e00583-23. https://doi.org/10.1128/spectrum.00583-23.

(25) Li, Q.; Xie, Y.; Cui, Z.; Huang, H.; Yang, C.; Yuan, B.; Shen, P.; Shi, C. Activation of Hypoxia-Inducible Factor 1 (Hif-1) Enhanced Bactericidal Effects of Macrophages to Mycobacterium Tuberculosis. *Tuberculosis* **2021**, *126*, 102044. https://doi.org/10.1016/j.tube.2020.102044.

(26) Schaaf, K.; Smith, S. R.; Duverger, A.; Wagner, F.; Wolschendorf, F.; Westfall, A. O.; Kutsch, O.; Sun, J. Mycobacterium Tuberculosis Exploits the PPM1A Signaling Pathway to Block Host Macrophage Apoptosis. *Sci Rep* **2017**, *7* (1), 42101. https://doi.org/10.1038/srep42101.
